# History of Adverse Pregnancy Outcomes, Blood Pressure, and Subclinical Vascular Measures in Late Midlife: SWAN (Study of Women's Health Across the Nation)

**DOI:** 10.1161/JAHA.117.007138

**Published:** 2017-12-29

**Authors:** Yamnia I. Cortés, Janet M. Catov, Maria Brooks, Siobán D. Harlow, Carmen R. Isasi, Elizabeth A. Jackson, Karen A. Matthews, Rebecca C. Thurston, Emma Barinas‐Mitchell

**Affiliations:** ^1^ Department of Epidemiology University of Pittsburgh Graduate School of Public Health Pittsburgh PA; ^2^ Departments of Obstetrics, Gynecology, and Reproductive Sciences and Epidemiology University of Pittsburgh School of Medicine Pittsburgh PA; ^3^ Department of Psychiatry University of Pittsburgh School of Medicine Pittsburgh PA; ^4^ Department of Magee Women's Research Institute Pittsburgh PA; ^5^ Department of Epidemiology University of Michigan School of Public Health Ann Arbor MI; ^6^ Department of Epidemiology and Population Health Albert Einstein College of Medicine Bronx NY; ^7^ Division of Cardiovascular Medicine Department of Internal Medicine University of Michigan Health Systems Ann Arbor MI

**Keywords:** blood pressure, cardiovascular disease, intima‐media thickness, pregnancy, Aging, Cardiovascular Disease, High Blood Pressure, Women

## Abstract

**Background:**

Adverse pregnancy outcomes, such as preterm birth (PTB), have been associated with elevated risk of maternal cardiovascular disease, but their effect on late midlife blood pressure (BP) and subclinical vascular measures remains understudied.

**Methods and Results:**

We conducted a cross‐sectional analysis with 1220 multiethnic parous women enrolled in SWAN (Study of Women's Health Across the Nation) to evaluate the impact of self‐reported history of adverse pregnancy outcomes (PTB, small‐for‐gestational‐age, stillbirth), on maternal BP, mean arterial pressure, and subclinical vascular measures (carotid intima‐media thickness, plaque, and pulse wave velocity) in late midlife. We also examined whether these associations were modified by race/ethnicity. Associations were tested in linear and logistic regression models adjusting for sociodemographics, reproductive factors, cardiovascular risk factors, and medications. Women were on average aged 60 years and 255 women reported a history of an adverse pregnancy outcome. In fully adjusted models, history of PTB was associated with higher BP (systolic: β=6.40; SE, 1.62 [*P*<0.0001] and diastolic: β=3.18; SE, 0.98 [*P*=0.001]) and mean arterial pressure (β=4.55; SE 1.13 [*P*<0.0001]). PTB was associated with lower intima‐media thickness, but not after excluding women with prevalent hypertension. There were no significant associations with other subclinical vascular measures.

**Conclusions:**

Findings suggest that history of PTB is associated with higher BP and mean arterial pressure in late midlife. Adverse pregnancy outcomes were not significantly related to subclinical cardiovascular disease when excluding women with prevalent hypertension. Future studies across the menopause transition may be important to assess the impact of adverse pregnancy outcomes on midlife progression of BP.

**Clinical Perspective**

**What Is New?**

- In a multiethnic population‐based study of women in late midlife, history of a preterm birth was significantly associated with higher blood pressure (BP) and mean arterial pressure, extending previous findings in premenopausal women.
- Even when excluding women with a history of hypertensive disorders of pregnancy, a prior preterm birth was associated with more adverse BP indices.
- History of adverse pregnancy outcomes (preterm birth, small for gestational age, stillbirth) were not significantly related to subclinical cardiovascular disease when excluding women with prevalent hypertension.

**What Are the Clinical Implications?**

- Findings suggest that women with a history of preterm birth exhibited a 6.4‐mm Hg higher systolic BP and a 3.2‐mm Hg higher diastolic BP compared with women with all term births. These data are clinically significant given that a 2‐mm Hg increment in systolic BP has been associated with a 7% increase in mortality from coronary artery disease and a 10% increase in mortality from stroke.
- Pregnancy history may help identify women who would benefit from cardiovascular risk assessment and modification. Proper monitoring and management of BP is warranted for women with a preterm birth.

## Introduction

Adverse pregnancy outcomes, including preterm birth (PTB; delivery<37 weeks’ gestation), small‐for‐gestational‐age birth (birthweight <10th percentile for gestational age), and stillbirth (pregnancy loss at ≥20 weeks) together affect ≈17% to 20% of births in the United States annually.^1^, ^2^ Accumulating evidence suggests that adverse pregnancy outcomes may serve as a screening test for future cardiovascular disease (CVD),^3^ the leading cause of morbidity and mortality in women.^4^ Prior studies report a 2‐ to 3‐fold increased risk of CVD in women with a history of PTB,^5^, ^6^, ^7^ even when not complicated by preeclampsia.^8^ In a record linkage study, severity and number of small‐for‐gestational‐age infants were associated with future maternal CVD‐related hospitalization or death (ie, coronary heart disease, cerebrovascular events, chronic heart failure).^9^ In studies examining pregnancy losses and CVD, women with a history of stillbirth had greater risk of subsequent coronary heart disease compared with women who had livebirths.^10^, ^11^ These data suggest that the underlying factors that contribute to women's risk for adverse pregnancy outcomes may also increase risk for CVD.^12^ Although the association between pregnancy‐associated hypertension and maternal CVD is well‐established,^13^, ^14^ previous studies have shown that low sensitivity may limit utility of maternal recall of hypertensive disorders of pregnancy.^15^

The majority of research relating adverse pregnancy outcomes to CVD is derived from small cohorts of relatively young women (mean age <50 years),^16^, ^17^ with low rates of CVD. Also, most are conducted in racially/ethnically homogeneous populations.^18^ Although racial/ethnic disparities exist in rates of adverse pregnancy outcomes and CVD,^19^, ^20^ racial differences in the association between adverse pregnancy outcomes and future risk of CVD has not been fully explored.^16^, ^21^ Furthermore, while the risk of CVD increases substantially after menopause,^22^ few studies have examined whether adverse pregnancy outcomes earlier in life influence women's CVD risk in midlife.^16^, ^23^, ^24^

Noninvasive measures of subclinical vascular disease including ultrasound assessment of carotid intima‐media thickness (IMT) and plaque burden and pulse wave velocity (PWV) measures of arterial stiffness are surrogate markers of arteriosclerosis and predictors of future cardiovascular events.^25^, ^26^ No studies have examined the impact of adverse pregnancy outcomes on subclinical CVD in late midlife, when subclinical disease significantly progresses.^27^ Elevated blood pressure (BP) is a critical risk factor for subclinical CVD in midlife.^28^ Recent data suggest that adverse pregnancy outcomes are associated with subsequent elevations in BP, and that BP may mediate associations between adverse pregnancy outcomes and future CVD.^24^, ^29^ Yet, prior studies did not consider whether these associations persist after women transition through menopause. Therefore, the purpose of the present analysis was to assess associations of adverse pregnancy outcomes (ie, PTB, small‐for‐gestational‐age infant, stillbirth) with BP, mean arterial pressure, and various indices of subclinical CVD in a large cohort of multiethnic women in late midlife. In doing so, we also sought to examine whether BP may be a potential pathway linking adverse pregnancy outcomes to subclinical CVD in late midlife. A secondary aim was to determine whether associations between adverse pregnancy outcomes and subclinical CVD were modified by race/ethnicity. A better understanding of the relationship between adverse pregnancy outcomes and cardiovascular health may lead to early identification of women at excess risk for CVD later in life.

## Methods

### Transparency and Reproducibility

SWAN (Study of Women's Health Across the Nation) provides access to public use data sets that extend through the tenth annual follow‐up visit. Some, but not all, of the data used for this manuscript are contained in the SWAN public use data sets.^30^ Investigators who require assistance accessing the public use data set may contact the SWAN Coordinating Center.

### Study Participants

SWAN is an ongoing longitudinal, multiethnic study of the biological and psychosocial changes that occur during the menopause transition. Details of the study design and recruitment have been described elsewhere.^31^ Briefly, between 1996 and 1997, a total of 3302 premenopausal or early perimenopausal women aged 42 to 52 years were enrolled at 1 of 7 research sites in Detroit, MI; Boston, MA; Chicago, IL; Oakland, CA; Los Angeles, CA; Newark, NJ; and Pittsburgh, PA. Baseline eligibility criteria for SWAN included: (1) an intact uterus and at least 1 ovary; (2) menstrual bleeding within the prior 3 months; (3) no current pregnancy or breastfeeding; and (4) no usage of reproductive hormones within the prior 3 months. Each site enrolled non‐Hispanic white women and women who self‐identified as 1 of 4 other predetermined racial/ethnic groups (black women in Detroit, Boston, Chicago, and Pittsburgh; Japanese women in Los Angeles; Chinese women in Oakland; and Hispanic women in Newark). Participants were assessed through a standardized protocol at study entry (in 1996–1997) and followed for an average of 14.3 years through 2011. Six sites (all sites except Los Angeles) assessed subclinical CVD using carotid ultrasound and PWV tests at visit 12 or visit 13.

Eligible women for the current analyses were those with at least 1 birth and who underwent a carotid scan or PWV assessment. Of 3302 women enrolled in SWAN, 2338 attended visit 13, 2249 of whom completed a pregnancy history questionnaire. Of these, 1854 provided information on 1 or more births (n=395 nulliparous). After excluding women without subclinical cardiovascular assessment at visits 12 or 13 (n=609) and those for whom small‐for‐gestational‐age birth could not be determined because of missing birth weight history (n=25), a total of 1220 women were included in this analysis. The institutional review boards at each study site approved the SWAN protocols. Each participant provided written informed consent.

### Exposure Variables

The primary exposure variables were reported history of PTB, term small‐for‐gestational‐age birth, and stillbirth. History of adverse pregnancy outcomes were assessed using a detailed interviewer‐administered pregnancy history questionnaire at SWAN visit 13 that collected information on number of births, birth outcomes (livebirth versus stillbirth), gestational age at delivery, and birth weight for each delivery. Interviews were conducted in the clinic/office, over the telephone, or in the respondent's home. Reported history of PTB was defined as a prior delivery at <37 completed weeks of gestation. A term small‐for‐gestational‐age birth (birthweight <10th percentile for ≥37 weeks’ gestational age) was determined using the World Health Organization weight percentile calculator,^32^ which uses a customized standard based on the sample mean birthweight at 40 weeks’ gestation.^33^ A history of stillbirth was considered as any pregnancy loss at ≥20 weeks’ gestation. Studies have shown high sensitivity (>0.90) and predictive validity for maternal recall of preterm delivery, small‐for‐gestational‐age birth, and pregnancy loss.^34^, ^35^ Women were categorized as having no adverse pregnancy outcomes, a single PTB, a term small‐for‐gestational‐age birth, a stillbirth, or multiple (>1) adverse pregnancy outcomes. Women with a preterm small‐for‐gestational‐age birth (n=4), which was defined as birthweight <10th percentile for <37 weeks’ gestational age, were included in the multiple adverse pregnancy outcome group.

### Outcome Variables

#### Blood pressure

BP measures in this analysis were collected at the time the carotid ultrasound was performed. BP was measured according to a standardized protocol, with readings taken on the right arm, with the respondent seated and feet flat on the floor for at least 5 minutes before measurement.^27^ The appropriate cuff size was determined based on arm circumference. A standard mercury sphygmomanometer was used to record systolic BP (SBP) and diastolic BP (DBP) at the first and fifth phase Korotkoff sounds. Respondents had not smoked or consumed any caffeinated beverages within 30 minutes of BP measurement. The average of 2 sequential BP values, with a minimum 2‐minute rest period between measures, was recorded. Using the average of these 2 sequential BP values, we calculated mean arterial pressure with a standard equation: [(SBP+2×DBP)/3].^36^

#### Brachial‐ankle PWV

Brachial‐ankle PWV (baPWV) was measured using the VP1000 system (Omron Healthcare), a noninvasive automated waveform analyzer. This device provides measures of baPWV, a measure of mixed central and peripheral PWV, on both the right and left sides, and the average of the 2 sides was used for our study. baPWV is the distance in centimeters between the brachial and ankle arterial recording sites divided by the time delay in seconds between the foot of the waveforms detected at the respective arterial sites. The distance or path length for brachial/ankle arterial sites was calculated based on a height‐based algorithm.^37^ The intra‐ and inter‐technician reliability was excellent with an intraclass correlation coefficient >0.93 for all sites. baPWV data were collected at visit 12 at the Pittsburgh site. Pittsburgh participants who did not have the baPWV test at visit 12 were tested at visit 13. baPWV was performed at visit 13 at other participating sites. As a result, baPWV data were available for 956 participants in this analysis.

#### Carotid ultrasound scan

Bilateral ultrasound carotid images were obtained using a Terason t3000 Ultrasound System (Teratech Corp) equipped with a variable frequency 5‐ to 12‐MHz linear array transducer. On each side, 2 digitized images were obtained of the distal common carotid artery, 1 cm proximal to the carotid bulb. From each of these 4 images, using the Artery Measurement System semiautomated edge detection software,^38^ IMT measures were obtained by electronically tracing and measuring the distance between the lumen‐intima and the media‐adventitia interfaces of the near and far walls of the common carotid artery. One measurement was generated for each pixel over the area, for a total of ≈140 measures for each image. The mean of the average readings of all 4 images were used in the analyses. Carotid scan images were read centrally at the SWAN Ultrasound Reading Center (University of Pittsburgh Ultrasound Research Lab).

The presence and extent of plaque were evaluated in each of 5 segments of the left and right carotid artery (distal and proximal common carotid artery, carotid bulb, and proximal internal and external carotid arteries).^39^ Consistent with the Mannheim and American Society of Echocardiography consensus statements,^40^, ^41^ plaque was defined as a distinct area protruding into the vessel lumen that was at least 50% thicker than the adjacent IMT and summarized as the presence or absence of any plaque. Additionally, for each of the bilateral carotid segments, the degree of plaque was graded between 0 (no observable plaque) to 3 (plaque covering ≥50% of the vessel diameter). The grades from all segments of the combined left and right carotid artery were summed to create the plaque index (possible range: 0–30).^42^ Sonographers at each study site were trained by the University of Pittsburgh Ultrasound Research Laboratory and monitored during the study period for reliability. Reproducibility for mean common carotid artery IMT measures was excellent, with an intraclass correlation coefficient between sonographers of 0.72 to 0.95, and between readers >0.87. The plaque index was found to be a valid and reproducible measure of carotid atherosclerosis in a number of populations (intraclass correlation coefficient, 0.86–0.93).^42^ The scanning and reading protocols have been used in numerous studies.^43^, ^44^

### Covariates

Self‐reported history of preeclampsia (high BP and proteinuria), gestational hypertension, and gestational diabetes mellitus (no diabetes mellitus prepregnancy) were also assessed at visit 13 using the detailed pregnancy history questionnaire. Previous studies have shown that maternal recall of hypertensive disorders of pregnancy has low sensitivity (yet high specificity, >90%) for all hypertensive disorders.^15^, ^45^ Studies have shown greater sensitivity and predictive validity for maternal recall of PTB, small‐for‐gestational age birth, and pregnancy loss.^34^, ^35^, ^46^ Therefore, relying on what is known about maternal self‐report, the primary exposures of interest in this analysis are PTB, small‐for‐gestational age birth, and stillbirth (pregnancy loss at ≥20 weeks). Because of the small sample of women with a reported history gestational diabetes mellitus, we did not consider it as a separate exposure. To address these limitations, sensitivity analyses were performed (as described in the Statistical Analysis) excluding women with a reported history of preeclampsia, gestational hypertension, and gestational diabetes mellitus.

Demographic and socioeconomic characteristics including race/ethnicity and education were assessed by self‐report at SWAN baseline. All other covariates were assessed at the time the carotid ultrasound was performed (corresponding to visit 12 or 13). Information on maternal age at first and last birth was assessed at visit 13 using the pregnancy history questionnaire for all women. Menopause status at the time of the carotid ultrasound measure was determined based on reports about frequency and regularity of menstrual bleeding and use of hormone therapy, as previously described.^47^ Current hormone use, including menopausal hormone therapy and oral contraceptives, was based on reported use since last SWAN visit.

Financial strain (ie, difficulty in paying for basics) was self‐reported and data from the visit corresponding to the carotid ultrasound was used in this analysis. Smoking (current/past, never), alcohol use, physical activity, and medication use (ie, antihypertensive, antidiabetics, lipid‐lowering) were drawn from the visit concurrent with the carotid ultrasound. Physical activity was assessed using modified Baecke Scores of Habitual Physical Activity, with higher scores indicating more physical activity.^48^ Body mass index was calculated as weight in kilograms divided by height in meters squared (kg/m^2^). Diabetes mellitus was defined as fasting glucose levels ≥126 mg/dL on ≥2 consecutive visits or any reported use of insulin/antidiabetic agents. Hypertension was defined as having a BP reading of ≥140/90 mm Hg or use of antihypertensive treatment.

Blood was drawn in the morning following a 12‐hour fast. Samples were frozen (−80°C) and sent on dry ice to the Medical Research Laboratories (Lexington). The homeostatic model assessment index was calculated from fasting insulin and glucose as (insulin [mU/L]×glucose [mmole/L per 22.5]).^49^ Triglycerides were analyzed by enzymatic methods on a Hitachi 747 analyzer (Boehringer Mannheim Diagnostics) and high‐density lipoprotein cholesterol was isolated using heparin‐2M manganese chloride.^50^ Low‐density lipoprotein cholesterol was calculated using the Friedewald equation.^51^

### Statistical Analysis

Variables were examined for distributions and outliers and transformation of data was applied as needed. To compare CVD risk factors across pregnancy outcome groups (no adverse pregnancy outcome, PTB, term small‐for‐gestational‐age birth, and more than 1 adverse outcome), ANOVA or Kruskal–Wallis tests were performed for continuous data and chi‐square or Fisher exact test for categorical variables. Post hoc analyses using the Dunnett test were conducted with the no adverse pregnancy outcome group as the reference group.

Associations between each adverse pregnancy outcome and each subclinical CVD measure were examined using multiple linear (BP indices, baPWV, IMT) and logistic regression (carotid plaque presence, plaque index) models. Models were first adjusted for age, race/ethnicity, site, financial strain, and age at first birth, with additional adjustments for covariates associated with adverse pregnancy categories and subclinical CVD measures at *P*<0.1. In analyses for baPWV, IMT, and plaque, models were next adjusted for SBP. Subsequent models adjusted for other traditional CVD risk factors (ie, body mass index, physical activity, smoking status, homeostatic model assessment of insulin resistance, and high‐ and low‐density lipoprotein cholesterol). Additional models adjusted for current use of antihypertensive, antidiabetic, and lipid‐lowering medications. Sensitivity analyses were also performed: (1) excluding women with prevalent hypertension (n=654), as defined earlier; (2) excluding women with a reported history of preeclampsia, gestational hypertension, or gestational diabetes mellitus (n=172); and (3) excluding women who were premenopausal/perimenopausal at the time of carotid ultrasound (n=74). Interactions between adverse pregnancy outcomes and race/ethnicity were examined by entering the appropriate cross product terms into multivariable models, and stratified analyses were performed for significant interactions. Residual analyses and model diagnostics were evaluated for evidence of collinearity in all models.

## Results

A total of 255 women (21%) reported a history of an adverse pregnancy outcome: 114 PTB, 59 term small‐for‐gestational‐age birth, 22 stillbirth, and 60 multiple adverse pregnancy outcomes (of which 44 had a PTB) (Table 1). At the time of the carotid scan visit, the women were an average age of 60 years, had some college education or more (51%), and 94% were postmenopausal. Post hoc analyses revealed that, compared with women who reported having no adverse pregnancy outcome, women who reported multiple adverse pregnancy outcomes were younger at first birth (22 years versus 26 years; *P*<0.001). The PTB group was more likely to report a history of preeclampsia, gestational hypertension, or gestational diabetes mellitus compared with those without a reported adverse pregnancy outcome (26% versus 12%; *P*<0.001). Rates of diabetes mellitus and hypertension at late midlife differed by adverse outcome group, with the lowest rates in the no adverse outcome group (Table 2). Mean±SD SBP and DBP at late midlife was highest for the multiple adverse outcome group (SBP: 131±19 mm Hg; DBP: 77.3±9.8 mm Hg), as was mean arterial pressure (95.4±11.9 mm Hg). Mean baPWV also differed by history of an adverse pregnancy outcome, with those reporting a prior PTB or term small‐for‐gestational‐age birth having higher baPWV than women with no adverse outcome. IMT was higher for the term for‐gestational‐age and multiple adverse outcome groups.

**Table 1:** Maternal Characteristics at Time of Subclinical CVD Assessment by Reported History of Adverse Pregnancy Outcomes (n=1220)

Variable	No Adverse Outcome (n=965)	PTB (n=114)	Term SGA (n=59)	Stillbirth (n=22)	>1 Adverse Outcome (n=60)	*P* Value^a^
Sociodemographics						
Age, mean±SD	60.3±2.7	60.0±2.8	60.1±2.4	59.7±2.7	59.8±3.1	0.04
Race/ethnicity, No. (%)						
White	464 (48.2)	57 (50.0)	17 (28.8)	11 (50.0)	12 (20.0)	<0.001^b^
Black	299 (31.1)	33 (29.0)	29 (49.2)	8 (36.4)	38 (63.3)^d^	
Hispanic	56 (5.8)	12 (10.5)^c^	5 (8.5)	2 (9.1)	8 (13.3)^d^	
Chinese	143 (14.9)	12 (10.5)	8 (13.6)	1 (4.6)	2 (3.3)	
Education						
<High school	75 (7.8)	11 (9.7)	6 (10.2)	1 (4.6)	8 (13.3)	0.09
Some college	475 (49.2)	59 (51.8)	34 (57.6)	11 (50.0)	37 (61.7)	
College degree/post college	415 (43.0)	44 (38.6)	19 (32.2)	10 (45.5)	15 (25.0)	
Financial strain (how hard to pay for basics), No. (%)						
Not hard at all	619 (64.6)	69 (60.5)	32 (54.2)	15 (68.2)	26 (43.3)	0.002
Somewhat/hard	339 (35.4)	45 (39.5)	27 (45.8)	7 (31.8)	34 (56.7)^c^	
Reproductive/pregnancy history						
Postmenopausal	915 (95.2)	105 (92.9)	55 (93.2)	19 (86.4)	52 (86.7)^c^	0.04^b^
Age at first birth, mean±SD, y	25.5±6.1	24.4±6.3	22.7±5.4^e^	24.1±5.6	21.9±5.3^e^	<0.001
Age at last birth, mean±SD, y	30.7±5.9	30.7±6.3	28.3±5.5^e^	32.5±4.9	28.9±5.5^e^	0.02
Parity, No. (%)						
1 or 2	612 (63.4)	55 (48.3)	34 (57.6)	5 (22.7)	24 (40.0)	<0.001^b^
3 or 4	314 (32.5)	48 (42.1)	21 (35.6)	14 (63.6)	29 (48.3)	
>4	39 (4.0)	11 (9.7)	4 (6.8)	3 (13.6)	7 (11.7)	
Hypertension or diabetes mellitus at pregnancy, No. (%)	119 (12.3)	29 (25.7)^e^	12 (20.3)	2 (10.0)	10 (16.7)	0.001^b^
Gestational hypertension/preeclampsia	85 (8.8)	21 (18.6)^e^	8 (13.6)	0 (0)	9 (15.0)	0.02^b^
Gestational diabetes mellitus	43 (4.5)	9 (8.0)	8 (13.6)^c^	2 (10.0)	2 (3.3)	0.02^b^

CVD indicates cardiovascular disease. Preterm birth (PTB)=delivery <37 weeks; term small‐for‐gestational‐age (SGA)=birth weight <10th percentile at 37 to 40 weeks’ gestation; stillbirth=pregnancy loss at ≥20 weeks’ gestation; >1 adverse pregnancy=any combination of the aforementioned outcomes, including a PTB (n=44), SGA (n=56), or stillbirth (n=9). Not all participants provided complete data. The actual number of observations per variable is noted when different from 1220.

a *P* value for overall group differences.

b Fisher's Exact Test performed excluding the “Stillbirth” group, given its small sample size.

c Post hoc analysis using Dunnett test differs from the no adverse pregnancy group (*P*<0.05).

d Post hoc analysis using Dunnett test differs from the no adverse pregnancy group (*P*<0.01).

e Post hoc analysis using Dunnett test differs from the no adverse pregnancy group (*P*<0.001).

**Table 2:** Cardiovascular Risk Factors and Subclinical CVD Outcomes at SWAN Visit 12 or 13 by Reported History of Adverse Birth Outcome (n=1220)

Variable	No Adverse Outcome (n=965)	PTB (n=114)	Term SGA (n=59)	Stillbirth (n=22)	>1 Adverse Outcome (n=60)	*P* Value^a^
Lifestyle factors						
Smoking status, No. (%) (n=1186)						
Never	862 (90.2)	96 (85.0)	44 (75.9)	22 (100.0)	52 (88.1)	0.007^b^
Past/current	94 (9.8)	17 (15.0)	14 (24.1)^d^	0 (0)	7 (11.9)	
Alcohol consumption, No. (%) (n=1172)						
<1 drink per mo	501 (53.0)	57 (51.8)	38 (67.9)	14 (63.6)	32 (53.3)	0.18
>1 drink per mo to <2 drinks per wk	241 (25.5)	29 (26.4)	6 (10.7)	5 (22.7)	20 (33.3)	
≥2 drinks per wk	204 (21.2)	24 (21.8)	12 (21.4)	3 (13.6)	8 (13.3)	
Physical activity score, mean±SD (n=1143)	7.6±1.8	7.6±1.9	7.0±1.8^c^	7.5±1.9	6.9±1.9^c^	0.001
Physical measures, chronic conditions, and current medications						
BMI, mean±SD, kg/m^2^ (n=1181)	30.0±7.1	29.9±7.9	31.4±8.7	32.3±7.5	31.8±7.8	0.01
Triglyceride, median (IQR) (n=1183), mg/dL	102 (75–138)	107 (81–144)	95 (75–142)	101 (85–119)	89 (69–145)	0.33
LDL‐C, mean±SD (n=1171), mg/dL	123.1±34.1	129.0±38.4	127.2±39.6	120.9±38.4	121.0±34.1	0.89
HDL‐C, median (IQR) (n=1176), mg/dL	59 (50–72)	60 (50–72)	59 (47–73)	56 (48–65)	58 (52–74)	0.79
HOMA‐IR, median (IQR) (n=1109)	2.16 (1.28–3.87)	1.93 (1.32–3.80)	2.38 (1.35–4.06)	3.85 (1.94–7.34)	3.26 (1.81–4.86)^d^	0.003
Systolic BP, mean±SD mm Hg	121.3±16.6	128.0±18.2	125.3±17.6	124.7±15.9	131.3±18.9	<0.0001
Diastolic BP, mean±SD mm Hg	73.5±9.4	76.8±11.1^d^	75.1±11.4	76.9±12.3	77.3±9.8^e^	0.0004
Mean arterial pressure, mean±SD, mm Hg	89.7±10.3	94.1±12.3	91.7±12.5	93.4±12.5	95.4±11.9	<0.0001
Hypertension, No. (%) (n=1166)	502 (53.3)	72 (65.5)^c^	35 (64.8)	13 (59.1)	45 (75.0)^d^	0.002
Diabetes mellitus, No. (%) (n=1187)	125 (13.1)	19 (17.0)	12 (21.1)	8 (36.4)^d^	14 (23.3)^c^	0.005^c^
Hormone therapy, No. (%) (n=1193)	39 (4.1)	3 (2.7)	4 (6.8)	1 (4.6)	3 (5.0)	0.62^b^
Antihypertensive treatment, No. (%) (n=1187)	382 (39.9)	54 (48.2)	27 (47.4)	10 (45.5)	32 (53.3)^c^	0.12
Antidiabetic therapy, No. (%) (n=1187)	102 (10.7)	18 (16.1)	9 (15.8)	6 (27.3)^c^	11 (18.3)	0.03
Lipid‐lowering treatment, No. (%) (n=1117)	279 (31.0)	40 (36.7)	18 (34.0)	6 (28.6)	13 (24.1)	0.55
Anticoagulants, No. (%) (n=916)	9 (1.3)	1 (1.1)	1 (2.3)	0	0	0.74
Subclinical CVD outcomes						
Brachial‐ankle PWV, mean±SD (n=956), cm/s	1227±213	1288±228^c^	1306±214	1291±311	1281±180	0.006
Average common carotid IMT, median (IQR)	0.78 (0.71–0.87)	0.78 (0.69–0.85)	0.80 (0.75–0.91)	0.76 (0.72–0.83)	0.81 (0.76–0.89)	0.04
Presence of plaque (yes), No. (%)	423 (43.8)	52 (45.6)	29 (49.2)	6 (27.3)	22 (36.7)	0.34

BMI indicates body mass index; BP, blood pressure; CVD, cardiovascular disease; HDL‐C, high‐density lipoprotein cholesterol; HOMA‐IR, homeostatic model assessment of insulin resistance; IMT, intima‐media thickness; IQR, interquartile range; LDL‐C, low‐density lipoprotein cholesterol; PWV, pulse wave velocity; SWAN, Study of Women's Health Across the Nation. Preterm birth (PTB)=delivery <37 weeks; term small‐for‐gestational‐age (SGA)=birth weight <10th percentile at 37 to 40 weeks’ gestation; stillbirth=pregnancy loss at ≥20 weeks gestation; >1 adverse pregnancy=any combination of the aforementioned outcomes, including a PTB (n=44), SGA (n=56), or stillbirth (n=9). Values derived from carotid scan visit or available visit nearest to carotid scan. Hypertension, diabetes mellitus, menopause status, and medication use reflects information provided at baseline through visit 12 or 13 (when the carotid scan was completed). Not all participants provided complete data. The actual number of observations per variable is noted when different from 1220.

a *P* value for overall group differences.

b Fisher exact test performed excluding the stillbirth group, given its small sample size.

c Post hoc analysis using Dunnett test differs from the no adverse pregnancy group (*P*<0.05).

d Post hoc analysis using Dunnett test differs from the no adverse pregnancy group (*P*<0.01).

e Post hoc analysis using Dunnett test differs from the no adverse pregnancy group (*P*<0.001).

In multiple linear regression analyses, a reported history of PTB or multiple adverse pregnancy outcomes was significantly associated with higher BP indices (Table 3). PTB remained significantly associated with all BP indices after excluding women with hypertension. Reported history of PTB or term small‐for‐gestational‐age birth was associated with higher baPWV in models adjusting for sociodemographics and age at first birth, but not in models adjusting for SBP (Table 4). We further adjusted for other CVD risk factors and medications, which did not significantly impact findings. Sensitivity analyses (excluding women with a reported history of preeclampsia, gestational hypertension, gestational diabetes mellitus, with exclusion of premenopausal/perimenopausal women) did not show any significant change in β coefficients (data not shown), although the association between PTB and baPWV approached significance when women with prevalent hypertension were excluded (*P*=0.09). No significant interactions were present between race/ethnicity and any adverse pregnancy outcomes for either BP or baPWV.

**Table 3:** Associations Between Reported History of Adverse Pregnancy Outcomes and BP at SWAN Visit 12 or 13

	SBP β (SE)	*P* Value	DBP β (SE)	*P* Value	MAP	*P* Value
PTB (any prior PTB vs no adverse birth outcome)						
Model 1 (adjusts for demographics and age at first birth)^b^	6.48 (1.65)	<0.0001	3.04 (0.96)	0.002	4.24 (1.12)	0.0002
Model 2 (model 1+CVD risk factors and medications)^c^	6.40 (1.62)	<0.0001	3.18 (0.98)	0.001	4.55 (1.13)	<0.0001
Model 3 (model 2+sensitivity analysis; n=538)^d^	5.03 (1.69)	0.003	2.68 (1.26)	0.03	3.46 (1.25)	0.006
Term SGA (any prior term SGA vs no adverse birth outcome)						
Model 1 (adjusts for demographics and age at first birth)^b^	2.36 (2.22)	0.29	1.16 (1.29)	0.37	1.03 (1.52)	0.50
Model 2 (model 1+CVD risk factors and medications)^c^	2.97 (2.42)	0.22	2.40 (1.44)	0.10	2.31 (1.68)	0.17
Model 3 (model 2+sensitivity analysis; n=517)^d^	1.10 (2.62)	0.66	1.23 (1.97)	0.53	1.19 (1.95)	0.54
>1 adverse pregnancy outcome (vs no adverse birth outcome)^a^						
Model 1 (adjusts for demographics and age at first birth)^b^	7.15 (2.37)	0.003	2.42 (1.34)	0.07	3.99 (1.49)	0.008
Model 2 (model 1+CVD risk factors and medications)^c^	7.30 (2.48)	0.003	2.30 (1.43)	0.11	3.97 (1.57)	0.01
Model 3 (model 2+sensitivity analysis; n=518)^d^	2.47 (2.41)	0.31	−1.36 (1.78)	0.45	−0.09 (1.77)	0.96

BP indicates blood pressure; CVD, cardiovascular disease; DBP, diastolic blood pressure; MAP, mean arterial pressure; PTB, preterm birth; SBP, systolic blood pressure; SGA, small‐for‐gestational‐age; SWAN, Study of Women's Health Across the Nation. Cross product of PTB*black, term SGA*black, and multiple adverse pregnancy outcomes*black tested for inclusion in each model and were nonsignificant (*P*≥0.05).

a Analysis limited to women with reported prior adverse pregnancy outcomes (n=60) vs. no adverse pregnancy outcome (n=754).

b Model 1 adjusted for site, age, race/ethnicity, financial strain, and age at first birth.

c Model 2: model 1 plus cardiovascular disease (CVD) risk factors (body mass index, physical activity, smoking, homeostatic model assessment of insulin resistance, and high‐ and low‐density lipoprotein).

d Model 3: model 2 plus sensitivity analysis excluding women with prevalent hypertension or antihypertensive treatment.

**Table 4:** Associations Between Reported History of Adverse Pregnancy Outcomes and baPWV at SWAN Visit 12 or 13

	β (SE)	*P* Value
PTB (any prior PTB vs no adverse pregnancy outcome)		
Model 1 (adjusts for demographics and age at first birth)^b^	55.5 (23.1)	0.02
Model 2 (model 1+SBP)^c^	12.8 (21.2)	0.54
Model 3 (model 2+CVD risk factors and medications)^d^	0.41 (22.2)	0.99
Model 4 (model 3+sensitivity analysis; n=435)^e^	51.4 (30.5)	0.09
Term SGA (any prior term SGA vs no adverse pregnancy outcome)		
Model 1 (adjusts for demographics and age at first birth)^b^	63.3 (31.0)	0.04
Model 2 (model 1+SBP)^c^	44.7 (28.5)	0.12
Model 3 (model 2+CVD risk factors and medications)^d^	12.9 (32.1)	0.69
Model 4 (model 3+sensitivity analyses; n=418)^e^	−22.1 (46.7)	0.64
>1 adverse pregnancy outcome (vs no adverse pregnancy outcome)^a^		
Model 1 (adjusts for demographics and age at first birth)^b^	28.8 (33.1)	0.38
Model 2 (model 1+SBP)^c^	−5.8 (30.1)	0.85
Model 3 (model 2+CVD risk factors and medications)^d^	−14.7 (32.6)	0.65
Model 4 (model 3+sensitivity analyses; n=419)^e^	−23.1 (42.0)	0.58

baPWV indicates brachial‐ankle pulse wave velocity; PTB, preterm birth; SGA, small‐for‐gestational‐age; SWAN, Study of Women's Health Across the Nation. Cross product of PTB*black, term SGA*black, and multiple adverse pregnancy outcomes*black tested for inclusion in each model and were nonsignificant (*P*≥0.05).

a Analysis limited to women with >1 birth; multiple adverse pregnancy outcomes (n=60) vs no adverse pregnancy outcome (n=754).

b Model 1 adjusted for site, age, race/ethnicity, financial strain, and age at first birth.

c Model 2: model 1 plus systolic blood pressure (SBP).

d Model 3: model 2 plus cardiovascular disease (CVD) risk factors (body mass index, physical activity, smoking, homeostatic model assessment of insulin resistance, and high‐ and low‐density lipoprotein).

e Model 4: model 3 plus sensitivity analysis excluding women with prevalent hypertension or antihypertensive treatment.

Table 5 presents results for the associations between adverse pregnancy outcomes and IMT. Reported history of PTB was associated with lower IMT in fully adjusted models. There was a significant interaction between history of PTB and race/ethnicity (*P*=0.006) in relation to IMT. Because of the small sample size of Hispanic and Chinese women, these analyses were limited to women who identified as black or white. In the fully adjusted models stratified by race/ethnicity, PTB was associated with lower IMT in black women but not in white women. When sensitivity analyses were performed excluding women with hypertension, PTB was not significantly associated with IMT and there was no significant interaction between history of PTB and race/ethnicity. Although term small‐for‐gestational‐age birth and multiple adverse pregnancy outcomes were associated with a higher IMT in unadjusted analyses, the association was attenuated when controlling for sociodemographic factors and age at first birth. Reported history of an adverse pregnancy outcome was not associated with carotid plaque presence or index, and these findings were not modified by race/ethnicity.

**Table 5:** Associations Between Reported History of Adverse Pregnancy Outcomes and IMT at SWAN Visit 12 or 13

	β (SE)	*P* Value
PTB (prior PTB only vs no adverse birth outcome)		
Model 1 (adjusts for demographics and age at first birth)^b^	−0.013 (0.012)	0.27
Model 2 (model 1+SBP)^b^	−0.027 (0.012)	0.02
Model 3 (model 2+CVD risk factors and medications)^d^	−0.025 (0.012)	0.04
Model 4 (model 3+sensitivity analysis; n=538)^e^	−0.011 (0.018)	0.54
Term SGA (prior term SGA only vs no birth pregnancy outcome)		
Model 1 (adjusts for demographics and age at first birth)^b^	0.031 (0.016)	0.06
Model 2 (model 1+SBP)^c^	0.029 (0.016)	0.07
Model 3 (model 2+CVD risk factors and medications)^d^	0.012 (0.018)	0.51
Model 4 (model 3+sensitivity analysis; n=517)^e^	0.009 (0.027)	0.74
>1 adverse pregnancy outcome (vs no adverse birth outcome)^a^		
Model 1 (adjusts for demographics and age at first birth)^a^	0.022 (0.017)	0.20
Model 2 (model 1+SBP)^b^	0.011 (0.017)	0.51
Model 3 (model 2+CVD risk factors and medications)^d^	0.003 (0.019)	0.87
Model 4 (model 3+sensitivity analysis; n=518)^e^	0.049 (0.025)	0.06

IMT indicates intima‐media thickness; PTB, preterm birth; SGA, small‐for‐gestational‐age; SWAN, Study of Women's Health Across the Nation. Cross product of PTB*black, term SGA*black, and multiple adverse pregnancy outcomes*black tested for inclusion in each model. Significant interaction for PTB (model 3: PTB*black=β −0.084, *P*=0.006; PTB=β 0.011, *P*=0.56).

a Analysis limited to women with >1 birth; multiple adverse pregnancy outcomes (n=60) vs no adverse pregnancy outcome (n=754).

b Model 2: model 1 plus systolic blood pressure (SBP).

c Model 1 adjusted for site, age, race/ethnicity, financial strain, and age at first birth.

d Model 3: model 2 plus cardiovascular disease (CVD) risk factors (body mass index, physical activity, smoking, homeostatic model assessment of insulin resistance, and high‐ and low‐density lipoprotein cholesterol).

e Model 4: model 3 plus sensitivity analysis excluding women with prevalent hypertension or antihypertensive treatment.

## Discussion

This is the first study in a racially diverse cohort of women in late midlife (aged 60 years) to assess the impact of PTB, term small‐for‐gestational‐age birth, and stillbirth on various indices of BP and subclinical CVD. History of a PTB was associated with higher indices of BP (SBP, DBP, and mean arterial pressure) but lower IMT in late midlife. For baPWV, the association was attenuated after adjusting for SBP. There was a significant interaction between PTB and race/ethnicity in relation to IMT, with PTB being associated with lower IMT in black women, but no significant relationship was found in white women. Moreover, there was no significant association between PTB and IMT when excluding women with hypertension or those taking antihypertensive treatment. Having multiple adverse pregnancy outcomes (a recurrent outcome or a combination of PTB, term small‐for‐gestational‐age birth, stillbirth) was associated with higher BP indices in fully adjusted models, but not after excluding women with prevalent hypertension. These associations did not differ by race/ethnicity.

This study was able to examine whether previous associations noted between PTB and BP^19^, ^27^, ^52^ remain significant in late midlife, when women transition through menopause and risk of CVD increases.^25^ Consistent with prior analyses, we found that history of PTB was positively related to BP even when excluding women with prevalent hypertension and preeclampsia, gestational hypertension, or gestational diabetes mellitus.^52^, ^53^ In fully adjusted models, we observed that women with a prior PTB exhibited 6.4 mm Hg higher SBP and 3.2 mm Hg higher DBP compared with women with all term births, a stronger association than that seen in previous studies,^19^, ^27^, ^52^ perhaps because of the older age of women in our sample. These data are clinically significant given that a 2‐mm Hg increment in SBP has been associated with a 7% increase in mortality from coronary artery disease and 10% increase in mortality from stroke.^54^ Mean arterial pressure, which has not been reported in prior studies of PTB and maternal CVD, was significantly higher among women with a history of PTB, indicating the potential impact of preterm delivery on overall blood flow and perfusion in late midlife. One explanation for this finding is that perhaps the women with PTB have a more vulnerable vasculature going into menopause (eg, more remodeling) and that the various cardiovascular challenges of menopause (eg, hormonal changes and body composition changes) and aging may thereby impact these women more adversely.^55^, ^56^ Studies have shown that modest elevations in BP, even within the normotensive range, contribute to risk of PTB.^57^ Thus, there may be small prepregnancy and during‐pregnancy differences in BP that are linked to PTB and increased BP later in life. However, the current analysis did not have prepregnancy data to examine this possibility. Our findings may further suggest that as women age, BP increases more rapidly among those with a history of PTB. Similarly, a history of multiple adverse pregnancy outcomes was associated with higher BP indices. We found a 7.3‐mm Hg higher SBP in women with multiple adverse pregnancy outcomes, suggesting that there may be a dose‐response relationship between number of adverse pregnancy outcomes and SBP in late midlife. However, the association was attenuated after excluding women with prevalent hypertension, perhaps attributable to a small sample size (75% of women in this group had hypertension). The most commonly reported adverse outcome in this group was PTB. Accordingly, history of PTB may help identify women at risk for higher BP in midlife and who may benefit from monitoring BP indices during the menopause transition. Although we were unable to differentiate between spontaneous (caused by premature rupture of membranes, premature labor, or cervical insufficiency) or medically indicated PTB, the exclusion of women with hypertensive disorders of pregnancy, gestational diabetes mellitus, and preterm small‐for‐gestational age births from this PTB group (the leading indications for medically inducted PTB), suggest that there is a common link between PTB and future maternal BP other than hypertension during pregnancy and this may extend to spontaneous PTB. Future studies with these clinical features, however, are needed to answer this important question.

Consistent with a previous study of subclinical CVD among women 4 to 12 years after pregnancy,^52^ our study found no significant association between PTB and baPWV after adjusting for SBP. One possibility is that baPWV, a combined measure of central and peripheral arterial stiffness,^58^ may be a less accurate measure of arterial stiffness than carotid‐femoral PWV.^59^ Although flow‐mediated dilation of the brachial artery is a consistent noninvasive measure predictive of long‐term cardiovascular events,^60^ this measure was only available in a subsample of women in our sample (n=376), of which ≈75 reported an adverse pregnancy outcome. Nonetheless, baPWV has been shown to increase with aging, hypertension, diabetes mellitus, and smoking.^61^ A borderline association was present when women with hypertension were excluded, indicating that BP may be an important factor in the association between PTB and arterial stiffness.

Our current finding that PTB is inversely associated with IMT in a cohort of mostly postmenopausal women, differs from that of a previous analysis in which women who delivered before 34 weeks’ gestation had higher IMT than those with term births, although this association was attenuated when adjusting for CVD risk factors.^52^ It is possible that our findings differ from this prior study because we did not have the adequate sample size to compare early (<34 weeks) and late (34–36) PTB. However, our study found that the association between PTB and IMT was modified by race/ethnicity. PTB was associated with lower IMT in black women but was not significantly associated with IMT in white women. Our stratified analyses found that black women with PTB were younger, had higher SBP, and reported greater rates of antihypertensive therapy than black women with no adverse pregnancy outcome. Hypertension induces dysfunctional alterations in the endothelium, which may result in thicker IMT.^62^ Antihypertensive treatment reduces progression of IMT,^63^, ^64^ potentially through functional or structural changes in the vessel wall.^65^ Excluding women with prevalent hypertension and antihypertensive medications from our analyses attenuated the negative association between PTB and IMT. Furthermore, there was no longer an interaction with race/ethnicity in these models. An assessment of IMT progression in a larger sample of women without hypertension would better characterize the impact of PTB on carotid remodeling in midlife.

Although not significant, it is important to note that a reported history of term small‐for‐gestational‐age birth and multiple adverse pregnancy outcomes was positively associated with IMT. Recent studies have also found a significant association between small‐for‐gestational‐age birth and BP,^24^, ^27^ although our data did not support this finding. However, our analysis had a smaller sample size, which may have impaired our ability to robustly detect differences between groups. It is also possible that sociodemographic characteristics not fully explained in our data underlie the association between small‐for‐gestational‐age birth and BP. Women with a history of term small‐for‐gestational‐age birth and multiple adverse pregnancy outcomes had higher body mass index and insulin resistance, suggesting that these pregnancy outcomes may lead to greater IMT through an association with metabolic factors.

In this analysis, we did not find an association between history of adverse pregnancy outcomes and carotid plaque, a finding consistent with related work among other samples of women.^66^ With less than half of our sample having any carotid plaque, sample size to examine this association was somewhat limited. Future work with samples of older women, women who are more likely to show plaque,^67^ can further investigate the association between adverse pregnancy outcome and carotid plaque.

### Study Limitations

There are several limitations to consider in this analysis. First, although the accuracy of maternal recall of preterm, small‐for‐gestational‐age birth, and stillbirth is high (>0.90),^16^, ^17^, ^18^ self‐reported history may still be a limitation. In addition, self‐report of preeclampsia and gestational hypertension may have been a limitation given the low sensitivity^15^, ^46^ of maternal recall of hypertensive disorders of pregnancy. Furthermore, our sample size of women with a term small‐for‐gestational‐age birth or stillbirth was smaller than in previous analyses^9^, ^10^, ^11^, ^27^, ^68^, ^69^ and may have limited our ability to detect an association between term small‐for gestation age birth and stillbirth with subclinical CVD. It is also possible that these adverse pregnancy outcomes may be related to CVD through another physiologic pathway (ie, socioeconomic drivers, body mass index, glucose dysregulation). In addition, data on prepregnancy CVD risk were not available, limiting our ability to determine whether differences in BP, lipid profile, or vascular measures were present before adverse pregnancy outcomes. Furthermore, although we excluded women with hypertensive disorders of pregnancy, gestational diabetes mellitus, and preterm small‐for gestational age from our models, we were unable to differentiate between spontaneous PTB and medically indicated PTB, which may have varying effects on maternal CVD risk. Future studies with clinical features of PTB are necessary to understand the impact of spontaneous versus medically indicated PTB on CVD in later life. Comparison between PTB subtypes is also necessary. For example, is there an association between early PTB (delivery <34 weeks) or very small‐for‐gestational‐age birth (birthweight <5th percentile for gestational age) and subclinical CVD in midlife? Previous reports support the potential for such associations.^27^, ^51^, ^68^ Last, we are unable to make definite conclusions regarding the magnitude of excess CVD risk in women in late midlife based on these measures of subclinical CVD alone. While the indices of subclinical CVD used in this study are good predictors of CVD,^70^, ^71^, ^72^ additional research is necessary to assess the impact of adverse pregnancy outcomes on endothelium‐dependent flow‐mediated dilation. Future studies with larger sample sizes may have the power to not only explore adverse pregnancy outcomes further but to examine severity of adverse pregnancy outcomes in relation to BP and subclinical CVD.

### Study Strengths

Strengths of this analysis include the relatively large sample of racially diverse women as well as the direct assessment of physical and vascular measures at late midlife. Our study provides information on the association between adverse pregnancy outcomes, BP, and subclinical CVD at late midlife, when absolute CVD risk increases.^25^ This study was able to examine whether the negative impact of adverse pregnancy outcomes on various indices of BP and subclinical CVD persist with aging. Furthermore, we examined the impact of having multiple prior adverse pregnancy outcomes, which has not been studied extensively and may pose a risk for later‐life CVD.

Although the American Heart Association now recognizes hypertensive disorders of pregnancy and gestational diabetes mellitus as a risk factor for future CVD and stroke,^73^ our findings suggest that history of PTB may be added to this group of pregnancy risk factors. With stillbirths and small‐for‐gestational‐age births accounting for 1% and 10% of births in the United States, respectively,^2^ additional studies about the association between these adverse pregnancy outcomes and CVD is necessary for early risk stratification and prevention. Although one of the strengths of this analysis was its focus on late midlife, when the overwhelming majority of women are postmenopausal, perhaps group differences may be detected in late perimenopause, when progression rates of subclinical CVD is greatest.^31^ Therefore, future research across the menopause transition may be important to determine the impact of adverse pregnancy outcomes on progression of BP and subclinical CVD.

## Conclusions

Our study shows that reported history of PTB is associated with higher BP indices in late midlife independent of prevalent hypertension and history of hypertensive disorders of pregnancy. A history of PTB was associated with lower IMT in black women and not white women, potentially because of the greater rate of hypertension in this group, as suggested by the attenuation of this association when excluding women with prevalent hypertension. With black women having excess rates of PTB, hypertension, and CVD,^74^, ^75^, ^76^, ^77^ there is a critical need to better understand racial/ethnic differences in the association between pregnancy‐related factors and progression of CVD. These findings suggest that history of PTB may help identify women with heightened BP in late midlife, a major contributor to CVD morbidity and mortality. In addition, this analysis demonstrates the importance of monitoring BP indices among women with a history of PTB.

## Sources of Funding

SWAN has grant support from the National Institutes of Health (NIH), Department of Health and Human Services, through the National Institute on Aging (NIA), the National Institute of Nursing Research (NINR), and the NIH Office of Research on Women's Health (ORWH) (grants U01NR004061; U01AG012505, U01AG012535, U01AG012531, U01AG012539, U01AG012546, U01AG012553, U01AG012554, U01AG012495). The content of this article is solely the responsibility of the authors and does not necessarily represent the official views of the NIA, NINR, ORWH, or the NIH. Cortés is supported by the Cardiovascular Epidemiology Training Program (T32HL083825).

## Disclosures

None.
